# The Study of Medicine

**Published:** 1902-09-06

**Authors:** 


### The Hospital

A Journal of

The Medical Sciences and Hospital Administration.

Vol. XXXII.—No. 832. Edited by Sir Henry Burdett, K.C.B., and Solomon C. Smith, M.D., M.R.C.P. [September 6, 1902.

### The Study of Medicine.

The first day of October, of this as of many preceding years, will witness the assembling together at our great hospitals, or at the schools of medicine connected with them, of large numbers of young men who are bent upon qualifying themselves to discharge the duties, and to reap the rewards, which are attendant upon the practice of the medical profession. But, while the day continues to be observed, the actual circumstances surrounding it have widely changed; insomuch that it may not be unprofitable to observe in what directions the changes are most manifest, and in what ways they require or suggest corresponding changes in the manner in which students should apply themselves to their work. In the middle of the century from which we have just emerged, to go back no farther than to a period well within the recollections of many who are still living and active, the student came to the school of medicine at the close of an apprenticeship, or term of domestic pupilage with a practitioner, during which, as his ability increased, he had been discharging more or fewer of the responsibilities of an assistant. He learned very little science, but he often acquired a considerable degree of clinical insight. His art was purely empirical, but his empiricism was of an eminently rational kind; and, in many of the daily demands of practice, he knew quite well what he was about. Upon such practical training would be grafted, during the next two and a half years, as much of the medical science of the period as could be conveniently compressed into them; and, to tell the truth, it would not amount to a great deal. Physiology, as we now understand it, was non-existent; pathology was limited to the recognition of gross changes; and medical chemistry aimed at little more than the detection of inorganic poisons in the viscera of a corpse.

In the present twentieth century all this has been altered, and the student now comes direct from school to the hospital, bringing with him no knowledge of the facts of illness, and very little of the manner in which disease modifies the characters of the men and women who are the subjects of it. He begins his studies without the smallest clue to their practical applications; without the power, for example, readily to appreciate the bearing of the anatomy of the skeleton upon the diagnosis of fractures, or the certainty derived from it as to the correct position of fragments. He has had no opportunity of acquiring that power of sympathy with patients which is so largely contributory to a correct estimation of their conditions; and a sick man appears to him chiefly in the light of a concrete illustration, useful to elucidate something which he has been taught. As for "science," he has only too much of it; too much, that is, in the sense of more than he can assimilate or apply. He suffers from a redundancy of text-books, little selected syllabuses picked out of the midst of various "ologies" on account of their presumed appropriateness to or bearing upon medicine; and in which, only too often, the same errors are copied by one compiler after another. He is in great danger of forgetting, or of never learning, that the object of medicine is not so much to cure diseases as to treat patients; and that, for this purpose, the patient must be studied as well as the disease. It is useless, of course, to think of reviving the system of fifty years ago, but it should not be suffered to pass into oblivion without due recognition of the benefits which it often conferred, or without urging upon the students of to-day the importance of doing all they can to secure benefits of the same kind through other channels. For this purpose they should seek every possible means of bringing themselves into close personal touch with patients, who, after all, may be described as the raw material out of which the successful practitioner manufactures reputation, and whom it is of primary importance to study and to understand. It follows that clinical clerkships, dresserships, and the like appointments should not only be diligently striven for, but should be used with constant reference to the establishment of friendly personal relations with the sufferers. There could be no more promising sign of the probable future success of the practitioner, than to see the faces of patients brighten, as the clinical clerk approaches their beds. The difference between the student who is in touch with patients and the student who is, so to speak, external to them is very conspicuous to a skilled examiner, and must often exert great influence in determining his verdict. A gracious word, a mere *nuance* of manner, will make all the difference between antagonism and accord, and the power to obtain the latter is one which will largely determine the measure of success to be expected in after life. Moreover, if we turn from the individual to the mass, from the student to the profession, it will largely determine the general estimation in which the medical body is held by the public, and the extent to which its influence will be operative in either public or professional questions.

